# A Genetic Screen for High Copy Number Suppressors of the Synthetic Lethality Between *elg1Δ* and *srs2Δ* in Yeast

**DOI:** 10.1534/g3.113.005561

**Published:** 2013-05-01

**Authors:** Inbal Gazy, Batia Liefshitz, Alex Bronstein, Oren Parnas, Nir Atias, Roded Sharan, Martin Kupiec

**Affiliations:** * Department of Molecular Microbiology and Biotechnology, Tel Aviv University, Ramat Aviv 69978, Israel; † Blavatnik School of Computer Science, Tel Aviv University, Ramat Aviv 69978, Israel

## Abstract

Elg1 and Srs2 are two proteins involved in maintaining genome stability in yeast. After DNA damage, the homotrimeric clamp PCNA, which provides stability and processivity to DNA polymerases and serves as a docking platform for DNA repair enzymes, undergoes modification by the ubiquitin-like molecule SUMO. PCNA SUMOylation helps recruit Srs2 and Elg1 to the replication fork. In the absence of Elg1, both SUMOylated PCNA and Srs2 accumulate at the chromatin fraction, indicating that Elg1 is required for removing SUMOylated PCNA and Srs2 from DNA. Despite this interaction, which suggests that the two proteins work together, double mutants *elg1Δ srs2Δ* have severely impaired growth as haploids and exhibit synergistic sensitivity to DNA damage and a synergistic increase in gene conversion. In addition, diploid *elg1Δ srs2Δ* double mutants are dead, which implies that an essential function in the cell requires at least one of the two gene products for survival. To gain information about this essential function, we have carried out a high copy number suppressor screen to search for genes that, when overexpressed, suppress the synthetic lethality between *elg1Δ* and *srs2Δ*. We report the identification of 36 such genes, which are enriched for functions related to DNA- and chromatin-binding, chromatin packaging and modification, and mRNA export from the nucleus.

Genome stability is of primary importance for the survival and proper functioning of all organisms. During DNA replication, the activity of DNA polymerases may be compromised by lesions or by the presence of secondary structures in the DNA. This may cause replication stalling and even fork collapse. Cells must react promptly to repair or bypass the DNA damage and to reactivate DNA replication. A number of mechanisms have been lately linked to this cellular response (Lee and Myung 2008).

PCNA is a homotrimeric ring that encircles the double-stranded DNA (Krishna *et al.* 1994) and plays a central role in DNA replication by ensuring the processivity of the replicative DNA polymerases (Chilkova *et al.* 2007; Krishna *et al.* 1994). Many proteins interact with PCNA and use it as a platform that enables stable interactions with DNA. These proteins include factors involved in DNA replication, DNA repair, chromatin remodeling, and other DNA-related activities that are important for cell viability, cell division, and genomic stability (Warbrick 2000).

In response to DNA damage, PCNA can be mono-ubiquitinated at lysine 164 by the E2/E3 pair Rad6 and Rad18 (Hoege *et al.* 2002). Mono-ubiquitination of PCNA allows the binding of the translesion synthesis polymerases to PCNA, resulting in an error-prone repair mechanism (Acharya *et al.* 2008). Alternatively, PCNA can be further poly-ubiquitinated on the same lysine residue by a mechanism that also requires Ubc13-Mms2 (E2 heterodimer) and Rad5 (E3) (Ulrich and Jentsch 2000). This poly-ubiquitination coordinates an error-free repair mechanism, whose details are still unclear (Branzei *et al.* 2008). Interestingly, the same residue of PCNA (lysine 164) can be modified by the ubiquitin-like molecule SUMO. This modification takes place during S-phase or after high doses of DNA damage. An additional residue, lysine 127, can also be SUMOylated but not ubiquitinated. In contrast to mutations in lysine 164, those in lysine 127 do not lead to DNA damage sensitive phenotypes (Hoege *et al.* 2002).

Two proteins have been shown to preferentially interact with SUMOylated PCNA in yeast: Srs2 and Elg1 (Armstrong *et al.* 2012; Kolesar *et al.* 2012; Parnas *et al.* 2010; Pfander *et al.* 2005). Srs2 is a DNA helicase; depending on the assay used, Srs2 has been shown to promote (Aylon *et al.* 2003) or prevent (Schiestl *et al.* 1990) homologous recombination. Srs2 was found to be able to displace the Rad51 strand-exchange protein (homologous to bacterial RecA) from ssDNA (Krejci *et al.* 2003; Veaute *et al.* 2003). SUMOylated PCNA recruits Srs2 to replication forks, where the helicase appears to prevent unscheduled recombination events (Papouli *et al.* 2005; Pfander *et al.* 2005).

The Elg1 protein resembles the large subunit of Replication Factor C, a protein complex in charge of loading/unloading PCNA from DNA during replication. Mutations in *ELG1* lead to a variety of genomic instability phenotypes, including, among others, hyper-recombination, chromosome loss, elongated telomeres, and increased telomeric silencing (Banerjee and Myung 2004; Banerjee *et al.* 2007; Bellaoui *et al.* 2003; Ben-Aroya *et al.* 2003; Kanellis *et al.* 2003). Similarly to Srs2, mutations in *ELG1* suppress the sensitivity to DNA damaging agents of mutants of the post-replication repair pathway (Parnas *et al.* 2010). Both proteins are recruited to SUMOylated PCNA by a similar mechanism involving PCNA- (PIP) and SUMO-interacting motifs (Armstrong *et al.* 2012; Kolesar *et al.* 2012; Parnas *et al.* 2010; Pfander *et al.* 2005). Moreover, deletion of *ELG1* leads to accumulation of Srs2 at the chromatin fraction, suggesting that Elg1 may play a role in Srs2 unloading, perhaps together with SUMOylated PCNA (Parnas *et al.* 2010). Remarkably, however, double deletion of the *ELG1* and *SRS2* genes results in a synthetic fitness reduction in haploid cells (Parnas *et al.* 2010) and in complete lethality in diploids (this study). These results imply that at least one of them must be active for viability. Because both proteins are active during DNA replication, it is likely that they provide alternative mechanisms to deal with specific DNA lesions or intermediates. However, alternative possibilities exist. Here we try to gain information about the function of these proteins by carrying out a genetic screen for genes that, when overexpressed, are able to rescue the synthetic lethality (SL) between *elg1* and *srs2* mutations.

## Materials and Methods

### Yeast strains

Strain OP738 (MATa/MATalpha elg1::KanMX/elg1:: KanMX srs2::KanMX srs2:: KanMX ade2/ade2 ade3/ade3 leu2/leu2 ura3/ura3 his3/his3 [p1313: CEN-LEU2-ELG1-ADE3]) was used for the genetic screen. Strain MK166 (lys2:: Ty1Sup ade2-1(o) can1-100(o) ura3-52 leu2-3, 112 his3del200 trp1del901 HIS3:: lys2:: ura3 his4:: TRP1:: his4) (Liefshitz *et al.* 1995) and its derivatives were used for testing sensitivity to DNA-damaging agents and to measure recombination levels.

### Genetic screen

A high copy number library in YEp24 (a 2-μm *URA3* plasmid) was used in the screen, carried out as described (Parnas *et al.* 2009). Each positive plasmid was extracted and retransformed into OP738. Only those that gave sectors were analyzed. Individual genes were subcloned into high copy number plasmids and retested.

GO (Gene Ontology) enrichment was calculated using an in-house score algorithm. The algorithm is a simple hypergeometric calculation; categories annotated with two few (≤3) or too many (≥500) genes were excluded from the enrichment calculation. Scores were corrected for multiple hypotheses testing an FDR threshold of 0.001.

## Results and Discussion

### SL between *elg1Δ* and *srs2Δ*

**Figure 1:**
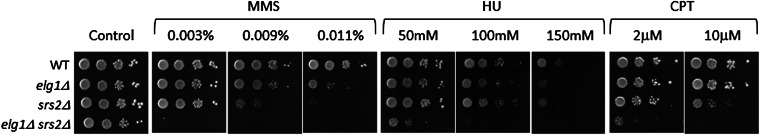
The *elg1Δ* and *srs2Δ* mutants display synergistic sensitivity to DNA-damaging agents. Ten-fold serial dilutions of logarithmic cultures were plated on SD-complete plates containing either methylmethane su\lfonate (MMS), hydroxyurea (HU), or camptothecin (CPT) at the concentrations shown.

Elg1 and Srs2 physically interact with SUMOylated PCNA in a similar manner through their PIP and SUMO-interacting domains (Armstrong *et al.* 2012; Kolesar *et al.* 2012; Parnas *et al.* 2010; Pfander *et al.* 2005). In addition, Elg1 seems to be required for the unloading of Srs2 from chromatin (Parnas *et al.* 2010). We would thus expect mutations in *ELG1* and *SRS2* to show epistasis, such that the double mutant would not show a phenotype more severe than the single mutants. However, *elg1Δ srs2Δ* double mutants show, in comparison with each of the single mutants, increased sensitivity to DNA-damaging agents, such as the alkylating agent methylmethane sulfonate, the inhibitor of deoxynucleotides synthesis hydroxyurea, and the topoisiomerase inhibitor camptothecin (Figure 1).

Mutations in each of the two genes cause an increase in homologous recombination (Ben-Aroya *et al.* 2003; Liefshitz *et al.* 1998). To characterize the type of recombination events that occur in the single and double mutants, we used strain MK166 (Liefshitz *et al.* 1995). This strain allows the detection of both gene conversion events between repeated sequences (Ty elements) or ectopic single sequences (*lys2* alleles) and direct-repeat recombination (DRR) at Ty elements and at the *HIS4* locus (Liefshitz *et al.* 1995). Whereas gene conversion transfers information between alleles, DRR results in the deletion of information located between the two repeats (Agmon *et al.* 2009). Table 1 shows that despite the fact that each mutant is hyperrecombinant, compared with the wt, they show a very different pattern of homologous recombination. Whereas *srs2Δ* shows increased levels of gene conversion events, the *elg1Δ* mutant shows both high gene conversion and high DRR levels. The *elg1Δ srs2Δ* double mutant shows the same level of DRR as *elg1Δ*; however, it exhibits a synergistic effect for gene conversion: *lys2* gene conversion is elevated eightfold, and the level of Ty ectopic conversion is increased 80-fold above that of the wt strain (Table 1). We interpret these results as follows: The activity of Srs2 prevents gene conversion events, probably by evicting the Rad51 recombination protein from the DNA (Krejci *et al.* 2003; Veaute *et al.* 2003). In the absence of Srs2, we see an increase in gene conversion events but not in DRR events, which are probably carried out by single strand annealing and do not require Rad51 (Krejci *et al.* 2003). In contrast, the absence of Elg1 promotes all types of recombination events, either because Elg1 does not allow the first steps of recombination to take place, or, as has been suggested (Davidson *et al.* 2012), because in its absence spontaneous DNA damage accumulates. The results of the double mutant, however, suggest that Elg1 activity restricts the level of gene conversion events of *srs2* mutants, and thus both proteins must play some role during the actual recombination process.

**Table 1:** Recombination frequency

Strain	His^+^ (DRR)	Ty DRR	Ty GC	Lys^+^ (GC)
wt	1 (10 × 10^−6^)	1 (1 × 10^−6^)	1 (0.5 × 10^−6^)	1 (0.25 × 10^−6^)
*srs2Δ*	1.1	1.1	3.5	2
*elg1Δ*	4.5	12	5	1.5
*elg1Δ srs2Δ*	4.4	11	80	8

Direct repeat recombination (DRR) is measured between tandem repeats at the *HIS4* locus or between Ty elements. Gene conversion (GC) measures the transfer of information between Ty elements or two copies of the *LYS2* gene carrying different mutations.

*elg1Δ srs2Δ* double mutants are extremely sick as haploids (Parnas *et al.* 2010), indicating that cells need at least one of the proteins to carry out a function important for fitness, probably some type of repair that is so common that it occurs often during DNA replication, such as fork stalling response. Previous work showed that many of the phenotypes of *srs2Δ* are stronger in diploids, and that several mutations affecting DNA repair produce a synthetic sick phenotype when combined with *srs2Δ* as haploids, but are completely lethal as diploids (Klein 2001). We thus crossed an *elg1Δ srs2Δ* haploid with either an *elg1Δ* haploid, an *srs2Δ* haploid or a double mutant *elg1Δ srs2Δ* of the opposite sex. Mating took place at normal frequencies. Zygotes were micromanipulated under the microscope to fixed positions in the plate and were then incubated for 48 hr. Thirty-two of 32 zygotes and 31 of 32 zygotes were able to form colonies in the first two crosses (with single *elg1Δ* and *srs2Δ* mutants, respectively), whereas only 2 of the 80 *elg1/elg1srs2/srs2* zygotes formed viable colonies after micromanipulation. Microscopic observation of the zygotes showed that the majority produced between 0 and 2 cell divisions before dying. Thus, diploid *elg1/elg1srs2/srs2* strains are inviable.

### A high copy number screen for suppressors of the SL between *elg1Δ* and *srs2Δ*

**Figure 2:**
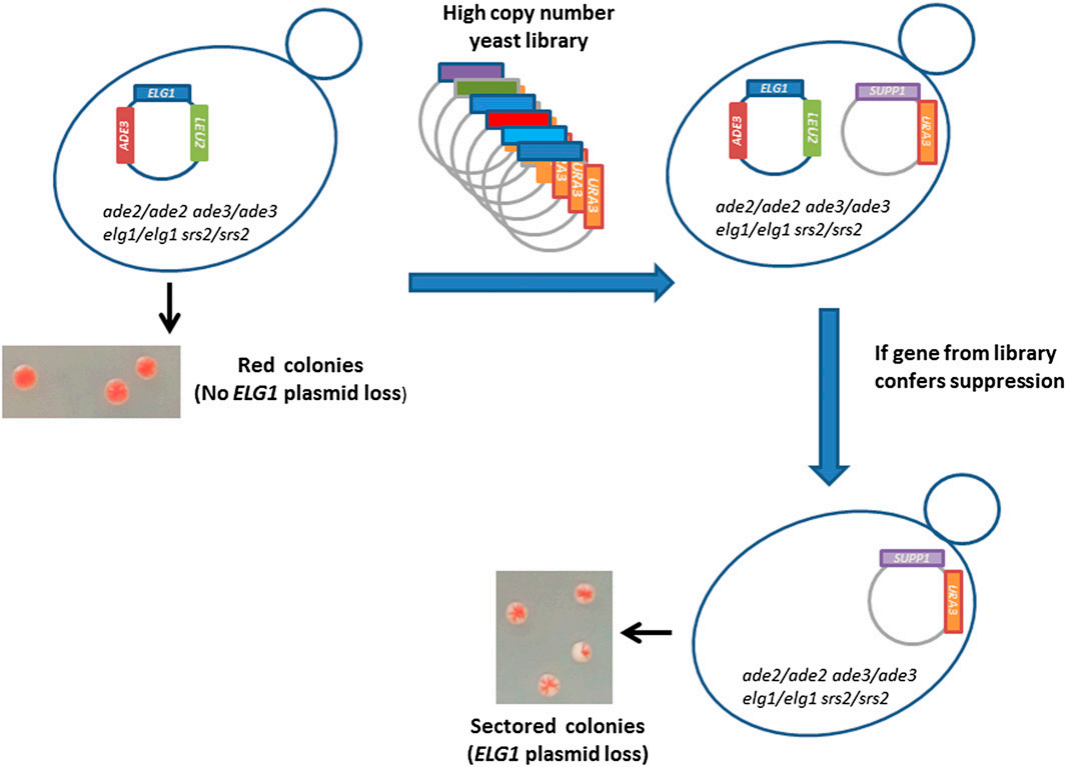
Schematic representation of the screen. A diploid double mutant *elg1Δ srs2Δ* strain is kept alive by the presence of a plasmid carrying the *ELG1* gene. The *ADE3* marker on the plasmid confers a red pigment to the cells carrying it (as the strain is *ade2 ade3)*. Since any cell that loses the plasmid during colony formation dies, all colonies are uniformly red. This strain was transformed with a high copy number library carrying random fragments of the yeast genome. Cells that received a plasmid that suppresses the SL phenotype (*SUPP1*) can now lose the *ELG1*-containing plasmid, becoming white. These cells create white or red/white sectored colonies.

**Figure 3:**
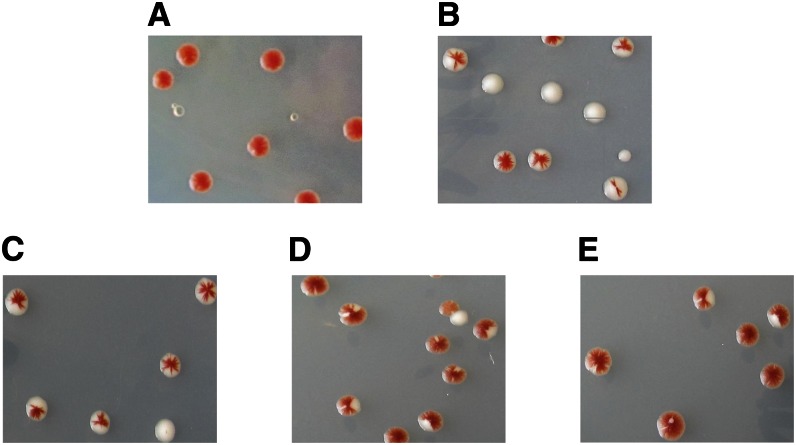
Examples of sectoring phenotypes observed in the presence of various high copy number plasmids. (A) Empty vector. (B) *ELG1*. (C) *MOB1* (strong suppressor). (D) *RTT109* (medium). (E) *PAB1* (weak suppressor).

Our high copy number screen was based on a red/white colony phenotype (Koren *et al.* 2003; Koshland *et al.* 1985; Parnas *et al.* 2009). *ade2* mutants form red colonies due to the accumulation of a red pigment. Mutations in the upstream-acting gene *ADE3* prevent the accumulation of the red pigment and render the cells white. An *ade2ade3elg1Δ srs2Δ* diploid strain (OP738) kept alive by the presence of a *LEU2*-marked plasmid carrying the *ELG1* gene as well as the *ADE3* gene forms uniformly red colonies (as the *ade3* mutation is complemented, and any cell that loses the plasmid dies; Figure 2). These cells were then transformed with a yeast genomic library cloned in a high copy number plasmid (containing the *URA3* marker). Cells that received a plasmid with a gene that, when overexpressed, can suppress the SL phenotype of *elg1Δ srs2Δ*, are now able to lose the *ELG1-ADE3-LEU2* plasmid, and therefore show white/red sectored colonies (Figures 2 and 3). The sectoring pattern (number and size of sectors) reflects the rate of viable plasmid loss, and thus provides a measure of the efficiency of suppression. Examples of such patterns are seen in Figure 3.

After screening ~50,000 yeast transformants, 92 candidates showed some level of colony sectoring. Plasmids (each containing a ~8 kb insert on average) were obtained from these colonies and retransformed into OP738. Only 54 of these candidates clearly exhibited sectors upon retransformation. These plasmids were sequenced to determine the genomic region responsible for the suppression. Many plasmids carried overlapping genomic fragments, and in total were found to represent 34 genomic regions. Subcloning experiments were then carried out to identify the genes responsible for the suppressive phenotype. With a few exceptions (*RTT103* and *NSE3*; *CHD1* and *PAB1*), only one gene per plasmid caused increased white sectoring. Table 2 shows the names and functions of the 36 genes identified.

**Table 2:** Genes in the screen

Gene Name	Short Description	Degree of Suppression
*BAP2*	High-affinity branched aminoacid permease	Strong
*ECO1*	Acetyltransferase required for Smc3p acetylation during the establishment of sister chromatid cohesion	Strong
*HTB1*	Histone H2B	Strong
*CDC34*	Ubiquitin-conjugating enzyme (E2) and catalytic subunit of SCF ubiquitin-protein ligase complex	Strong
*CHD1*	Chromatin remodeller	Strong
*CRM1*	Major karyopherin, involved in export of proteins, RNAs, and ribosomal subunits from the nucleus	Strong
*MOB1*	Component of the mitotic exit network	Strong
*DDC1*	Member of the 9-1-1 clamp essential for the DNA damage response	Strong
*CST6*	Basic leucine zipper (bZIP) transcription factor	Strong
*MPH1*	Helicase that functions in an error-free DNA damage bypass pathway	Strong
*YKL044w*	Gene of unknown function	Strong
*SIS2*	Negative regulatory subunit of the protein phosphatase 1 Ppz1	Strong
*UBC11*	Ubiquitin-conjugating enzyme	Strong
*NHP6A*	High-mobility group non-histone chromatin protein	Strong
*TPT1*	tRNA 2’-phosphotransferase, catalyzes the final step in yeast tRNA splicing	Strong
*TAF3*	TFIID subunit involved in transcription initiation	Strong
*RTT103*	Involved in transcription termination	Medium
*SRL3*	Cytoplasmic protein that, when overexpressed, suppresses the lethality of a rad53 null mutation	Medium
*RTT109*	Histone H3 acetylase	Medium
*GID8*	Member of a ubiquitin ligase that carries out poly-ubiquitination.	Medium
*GIS2*	Protein with seven cysteine-rich CCHC zinc-finger motifs	Medium
*RTT106*	Histone chaperone	Medium
*YOL114c*	Gene of unknown function	Medium
*IRC13*	Null mutant displays increased levels of spontaneous Rad52 foci	Medium
*NUP1*	Nuclear pore complex (NPC) subunit, involved in protein import/export and in export of RNAs	Weak
*PPS1*	Protein phosphatase with specificity for serine, threonine, and tyrosine	Weak
*UBP14*	Ubiquitin-specific protease	Weak
*NSE3*	Essential subunit of the Mms21-Smc5-Smc6 complex	Weak
*PAB1*	Poly(A) binding protein, part of the 3′-end RNA-processing complex	Weak
*DAM1*	Essential subunit of the Dam1 complex, involved in kinetochore movement	Weak
*CPD1*	Cyclic nucleotide phosphodiesterase	Weak
*UBP7*	Ubiquitin-specific protease that cleaves ubiquitin-protein fusions	Weak
*RPB4*	Subunit of RNA polymerase involved in 3′-end RNA processing and export	Weak
*RFX1*	Major transcriptional repressor of DNA-damage-regulated genes	Weak
*HAS1*	ATP-dependent RNA helicase, localizes to nuclear pore	Weak
*CNM67*	Component of the spindle pole body outer plaque	Weak

The genes identified span a large number of functions, pathways and cellular location. Analysis of GO (gene onthology) term enrichment (Table 3) shows that this gene set is enriched for genes of chromosomal location, with functions related to DNA- and chromatin-binding and involved in biological processes related to DNA packaging and modification. Additionally, there is an enrichment for genes involved in regulatory mechanisms as well as genes that participate in the response to DNA damage. Below, we present the genes, divided into functional categories obtained by reading the literature regarding each of the genes. Naturally, this division is artificial, as many of the categories overlap, and the precise mechanism of suppression is still unknown.

**Table 3:** GO annotation enrichment

Description	GOID	Ontology	Intersection	Category Size	*P* Value
Chromosome	GO:0005694	Cellular compartment	11	393	2.33E-06
Chromosomal part	GO:0044427	Cellular compartment	9	352	4.78E-05
Structure-specific DNA binding	GO:0043566	Molecular function	5	101	1.47E-04
Chromatin binding	GO:0003682	Molecular function	4	84	8.27E-04
Regulation of mitotic cell cycle	GO:0007346	Biological process	6	105	1.33E-05
Negative regulation of cellular process	GO:0048523	Biological process	10	422	3.17E-05
Negative regulation of biological process	GO:0048519	Biological process	10	432	3.86E-05
DNA repair	GO:0006281	Biological process	8	263	4.05E-05
DNA packaging	GO:0006323	Biological process	4	61	2.49E-04
Chromatin assembly or disassembly	GO:0006333	Biological process	4	66	3.36E-04
Nucleosome organization	GO:0034728	Biological process	4	69	3.97E-04
DNA conformation change	GO:0071103	Biological process	4	80	6.91E-04
Negative regulation of transposition	GO:0010526	Biological process	2	8	7.22E-04
Response to DNA damage stimulus	GO:0006974	Biological process	7	307	7.27E-04
Negative regulation of cellular metabolic process	GO:0031324	Biological process	7	307	7.27E-04
DNA replication-independent nucleosome organization	GO:0034724	Biological process	2	9	9.23E-04

An FDR correction of 0.001 was used

#### Genes affecting genome stability:

This category includes the following genes:

##### ECO1/CTF7:

Eco1 is a protein essential for sister chromatid cohesion (Skibbens *et al.* 1999; Toth *et al.* 1999). It has an acetyltransferase activity that acetylates the cohesin subunit Smc3. This acetylation has been shown to be critical for the establishment of cohesion during S phase (Unal *et al.* 2008). Eco1 interacts with PCNA through a PIP motif (Moldovan *et al.* 2006), thus effectively coordinating between cohesion establishment and DNA replication. It was found that loss of PCNA SUMOylation by deletion of the SUMO ligase *SIZ1* rescued the temperature sensitivity of *eco1* mutants, indicating a role for SUMO in this process (Moldovan *et al.* 2006). Elg1 has been shown to affect sister chromatid cohesion (Parnas *et al.* 2009) and genetic antagonistic interactions have been observed between Elg1 and Eco1: deletion of *ELG1* partially rescues the phenotypes of a temperature sensitive *eco1* mutant, whereas its overexpression enhances its phenotypes (Maradeo and Skibbens 2009; Maradeo and Skibbens 2010). Despite their common interaction with PCNA, no physical or genetic interactions have been detected between Eco1 and Srs2.

##### NSE3:

Nse3 is part of the Smc5-6 complex, which is structurally similar to cohesin, but whose precise function still remains enigmatic (Bustard *et al.* 2012). This complex was found to be required for a variety of DNA repair activities, including sister chromatid recombination, and mutants defective for its components exhibit an unusual amount of recombination intermediates (Bustard *et al.* 2012), a phenotype shared with both *elg1Δ* and *srs2Δ* mutants. Interestingly, the complex contains a SUMO-ligase and potentially a ubiquitin-ligase. A recent publication links cohesin SUMOylation by the Smc5-6 complex to cohesion establishment, in an Eco1-independent fashion (Almedawar *et al.* 2012).

##### MPH1:

Mph1 is the yeast homolog of FANCM, a human gene that, when mutated, leads to Fanconi anemia. Mph1 is a DNA helicase involved in error-free bypass of DNA lesions. It has been proposed that Mph1 may promote sister chromatid recombination as a way of bypassing lesions that stall fork progression (Ede *et al.* 2011). Interestingly, deletion of *MPH1* rescues the DNA damage sensitivity of mutants of the Smc5-6 complex (Chavez *et al.* 2011; Chen *et al.* 2009), whereas Mph1 overexpression exacerbates the mutants’ phenotypes (Chen *et al.* 2009). Physical and negative genetic interactions have been observed between Mph1 and Srs2 (Chiolo *et al.* 2005; St Onge *et al.* 2007). *mph1* mutants share with both *elg1* and *srs2* genome instability phenotypes (Daee *et al.* 2012), suggesting that Mph1 may be sharing repair functions with both genes.

##### DDC1:

Ddc1 forms part of a PCNA-like molecule (called the 9-1-1 clamp). Loading of this heterotrimeric ring onto DNA is essential for the activation of the DNA damage response (Melo *et al.* 2001). This clamp has been recently been proposed to play a pivotal role in an error-free lesion bypassing mechanism that operates in parallel to Srs2 (Karras *et al.* 2013).

These four genes seem to be exerting their suppressive effect by affecting processes that take place during DNA replication, at the fork. Their overexpression may prevent stalling or may be able to act in DNA repair, either replacing a common function of Elg1 and Srs2, or providing alternative repair mechanisms. Ddc1 overexpression may lead to a stronger or extended DNA checkpoint response, which may help cope with fork stalling. Alternatively, the lethality between elg1 and srs2 may be due to lesions left behind the fork in the absence of the two repair activities, and overexpression of proteins that participate in alternative repair mechanisms may be able to repair this lethal damage, rescuing the cells.

#### Genes affecting histones and chromatin:

This category includes the following genes:

##### HTB1:

DNA is packaged in eukaryotic cells around nucleosomes composed of two units each of the H2A-H2B and H3-H4 heterodimers. *HTB1* encodes a copy of histone H2B. Ubiquitination of this histone on residue K123 is a prerequisite for histone H3 modification and is essential for a variety of cellular processes, including gene expression and DNA repair (Dover *et al.* 2002; Robzyk *et al.* 2000).

##### RTT106:

During DNA replication, nucleosomes ahead of the replication fork are disassembled, and the recently replicated DNA is reassembled into nucleosomes using both the old, parental histones, as well as newly synthesized histones. Deposition of newly synthesized H3–H4 requires histone chaperones, including Rtt106 (Li *et al.* 2012). The dimeric histones delivered by Rtt106 are marked by acetylation at lysine 56 of histone H3 (Su *et al.* 2012).

##### RTT109:

Rtt109 is a histone acetyltransferase that is required for proper acetylation of histone H3 at lysine 56 (H3K56), which occurs during S phase and DNA damage repair. The formation of H3K56ac by Rtt109 and its deposition by Rtt106 stabilize the advancing replication forks and allow the repair of lesions that occur during replication (Clemente-Ruiz *et al.* 2011).

##### CHD1:

Chd1 is a chromatin remodeler that belongs to the chromodomain-containing subfamily of Snf2-like proteins; these proteins are believed to be recruited through methylated histone lysine residues and to promote nucleosome remodeling. Interestingly, it was recently shown that Chd1 is required for histone H2B ubiquitination, which is required for histone restoration after RNA polymerase passage (Lee *et al.* 2012).

##### NHP6A:

Nhp6A is an abundant small protein that binds DNA nonspecifically and bends it sharply. By binding the minor groove of DNA with a single HMGB domain and wrapping around the DNA to contact the major groove, it helps remodeling the nucleosomes (Stillman 2010). A less-abundant, very similar protein, Nhp6B, exists. Both were found to be components, together with Spt16p and Pob3p, of the FACT complex, which plays important roles in transcription and DNA replication (Ruone *et al.* 2003).

Thus, overexpression of histone H2B, or of proteins that promote nucleosome re-establishment after DNA or RNA polymerase transit, alleviate the SL phenotype of *elg1Δ* and *srs2Δ*. We can think of three possible models for this: (1) mutations in these genes cause replication fork instability, which can be suppressed by accelerating replication in the presence of excess histone dimers ready to be assembled into the newly replicated DNA. (2) Alternatively, Srs2 and Elg1 may play a direct role in the regulation of histone levels in the cell, and their SL is alleviated when histone levels are increased by other means. (3) A third model, involving an effect of these chromatin regulators in transcription regulation (which is also affected by histone levels and deposition), seems to us less likely, although recent evidence supports a role for histone levels in the regulation of promoter fidelity (Silva *et al.* 2012).

It is interesting to note that *elg1* mutants are synthetic sick with mutations in *HTB1* (data not shown), *RTT106* (Imbeault *et al.* 2008), *RTT109* (Bellaoui *et al.* 2003), although not with *chd1Δ*, *nhp6aΔ* or the double *nhp6aΔ nhp6bΔ* (data not shown). In contrast, *srs2Δ* shows a synthetic fitness phenotype when combined with *chd1Δ* (Pan *et al.* 2006), *rtt109Δ* (Costanzo *et al.* 2010), but not with *htb1Δ*, *rtt106Δ*, *nhp6aΔ* or the double *nhp6aΔ nhp6bΔ* (data not shown). Thus, the spectrum of synthetic phenotypes between *elg1Δ* and *srs2Δ* only partially overlap, consistent with nonoverlapping functions for the two genes.

#### Genes affecting RNA end-processing and/or nuclear transport:

This category includes the following genes:

##### RTT103:

Transcription by RNA polymerase II (PolII) and transcript processing are coordinated by the phosphorylation status of the PolII C-terminal domain (CTD). Transcription termination requires, surprisingly, the activity of RNA nucleases such as Rat1, which is recruited by Rtt103 (Kim *et al.* 2004). This requires an interaction between Rtt103 and the Ser2-phosphorylated form of PolII CTD. It was recently found that phosphorylation of the Tyr-1 residue in the CTD prevents Rtt103 binding, thus enhancing transcription processivity. At the 3′ end of the transcript Tyr-1 levels drop, allowing Rtt103 binding and transcription termination followed by poly-adenylation (Mayer *et al.* 2012).

##### PAB1:

Pab1 associates with RNA poly(A) tails, mediating the interactions between the 5′ cap structure and the 3′ mRNA poly(A) tail. It plays a role in mRNA stability and stimulates the initiation of translation (Amrani *et al.* 2008; Amrani *et al.* 1997). In addition, Pab1 is essential in the coupling of 3′ RNA processing and nuclear export of the mRNA (Dunn *et al.* 2005).

##### RPB4:

Rpb4 is an RNA polymerase II subunit that forms a complex with Rpb7. Contrary to other subunits of RNA polymerase II, Rpb4 is not essential under normal conditions; however, it becomes essential under stress. Lately it was found that this complex plays an important role in coordinating transcription in the nucleus with mRNA degradation and translation in the cytoplasm (Harel-Sharvit *et al.* 2010).

##### GIS2:

Gis2 binds to a subset of mRNA molecules and promotes their translation by a still poorly understood mechanism (Sammons *et al.* 2011; Tsvetanova *et al.* 2010). Both Gis2 and its human ortholog, CNBP, mutations that cause muscular dystrophy, are part of stress granules (Rojas *et al.* 2012).

##### HAS1:

The *HAS1* gene encodes an ATP-dependent RNA helicase that plays an important role in ribosomal RNA processing and nuclear export. Accordingly, it is highly enriched in nuclear pore complex fractions (Rout *et al.* 2000).

##### NUP1:

The Nup1 protein is a component of the central core of the nuclear pore complex. It plays a role in RNA and protein transport through the pore, by serving as a release factor for karyopherin (Aitchison and Rout 2012; Izaurralde and Adam 1998).

##### CRM1:

Crm1 is the main karyopherin protein, involved in export of proteins, RNAs, and ribosomal subunits from the nucleus. It plays a central role in cargo export (exportin function) (Aitchison and Rout 2012).

The common mechanism suggested by these genes is that the SL phenotype observed can be suppressed by increasing the levels of certain mRNA(s) as well as their transport from the nucleus (through the nuclear pore), perhaps causing an increase in the translation level of (a) target gene(s).

#### Spindle pole body and spindle checkpoints:

##### CNM67:

Cnm67 is a component of the spindle pole body outer plaque. It plays a central role in spindle orientation and in mitotic nuclear migration (Brachat *et al.* 1998). Cnm67 interacts physically and genetically with Gis2 (Scherrer *et al.* 2011; Wilmes *et al.* 2008). It also interacts with several kinetochore components, and mutations in *CNM67* exhibit a synthetic fitness defect when combined with *srs2* mutations (Pan *et al.* 2006).

##### DAM1:

Dam1 is a member of the DASH complex, which facilitates microtubule-kinetochore interactions, thereby playing an essential role in chromosome segregation. The DASH complex forms a ring around microtubules that stabilizes the microtubule and promotes its growth (Miranda *et al.* 2005). It also allows the kinetochore to preferentially bind to the microtubule plus end (Miranda *et al.* 2005). Dam1 is phosphorylated by the Ipl1 Aurora kinase; although this phosphorylartion does not seem to affect the binding activities of the complex, it is essential for the establishment of bipolar attachments. Notably, Dam1 is also methylated by the Set1 methyltransferase, the same enzyme in charge of methylating histone H3 at lysine4 (H3K4). Remarkably, both H3K4 and Dam1 methylation depend on prior ubiquitination of histone H2B (Latham *et al.* 2011). Mutations in *DAM1* show synthetic fitness defects with mutations in *RTT103*.

##### MOB1:

Mob1 is a member of the mitotic exit network. It is located at the spindle pole body, where it binds and regulates the Dbf2 protein kinase. Activation by Cdc15p-dependent phosphorylation leads to a change in its location, inducing cytokinesis and cell separation (Mah *et al.* 2001). Interestingly, Mob1 is important for the proper localization of the Ipl1 Aurora kinase (Stoepel *et al.* 2005), which phosphorylates Dam1. Thus, mitotic exit network activity is important for proper kinetochore activity, and controls the spindle checkpoint (Tan *et al.* 2005).

The mechanism by which these genes may suppress the SL of *elg1Δ srs2Δ* cells could be related to the spindle checkpoint, which gets activated as a secondary response to DNA damage and prevents adaptation to DNA damage in *srs2Δ* mutants (Dotiwala *et al.* 2010).

#### Protein modification:

A number of genes encoding protein modifiers were identified; these include ubiquitin-related proteins, as well as a protein phosphatase and a regulator of another phosphatase. The targets of these proteins that allow suppression of the SL phenotype is still unclear:

##### CDC34:

Cdc34 encodes a ubiquitin-conjugating enzyme (E2) that is the catalytic subunit of the SCF ubiquitin-protein ligase complex. This complex is in charge of targeting proteins for degradation, particularly those involved in cell cycle progression. Cdc34 levels are increased under conditions of DNA replication stress, suggesting that it plays an important role in coping with this type of genotoxic situation (Tkach *et al.* 2012).

##### UBC11:

Ubc11 is a ubiquitin-conjugating enzyme (E2). Its E3 partners, targets or functions are currently unknown.

##### GID8:

Gid8 is part of another multiprotein complex that functions as an E3 ubiquitin ligase to ubiquitinate proteins to send them to degradation by the proteasome (Menssen *et al.* 2012).

##### UBP14:

Ubp14 is a deubiquitinating protein that disassembles free poly-ubiquitin chains, thus controlling the rate of degradation by the proteasome (Amerik *et al.* 1997). Interestingly, Ubp14 collaborates with the Gid complex (Regelmann *et al.* 2003) and mutations in this gene are synthetic lethal with mutations in Cdc34 (Cocklin *et al.* 2011).

##### UBP7:

Ubp7 is another deubiquitination enzyme of unknown function.

##### PPS1:

Pps1 is a phosphatase that plays a role during S-phase (Ernsting and Dixon 1997). Its targets remain unknown.

##### SIS2:

Sis2/Hal3 binds to the C-terminal catalytic domain of the serine/threonine phosphatase Ppz1 and strongly inhibits its activity (de Nadal *et al.* 1998). Decreased activity of Ppz1 leads to salt tolerance, compromised cell integrity and accelerated G1/S transition (Munoz *et al.* 2003; Posas *et al.* 1995). In addition, *SIS2/HAL3* has a role in the biosynthetic pathway of coenzyme A, as part of a different complex, the PPCDC (phosphopantothenoylcysteine decarboxylase complex), which also includes the Cab3 and Vhs3 subunits (Ruiz *et al.* 2009). However, transformation of the *elg1Δ srs2Δ* strain with plasmids overexpressing these two subunits conferred no suppression. We thus conclude that it is likely that its interaction with Ppz1 phosphatase is the one responsible for the suppression (although we cannot rule out the possibility that Sis2, and only Sis2, is the limiting factor in the activity of the CoA synthesis complex). Overexpression of a phosphatase repressor may increase the level of a phosphorylated protein, which may be limiting in the *elg1Δ srs2Δ* strains.

#### tRNA splicing:

Two genes were found, encoding successive steps in tRNA splicing.

##### TPT1:

*TPT1* is an essential gene that encodes tRNA 2′-phosphotransferase, which catalyzes the final step of tRNA splicing (Culver *et al.* 1997). tRNA splicing is carried out by a multisubunit endonuclease, followed by ligation. Tpt1 catalyzes the transfer of the splice junction 2’-phosphate from the ligated tRNA to NAD to produce ADP-ribose 1′′-2′′ cyclic phosphate (Appr > p) (Culver *et al.* 1997). In a conditional lethal *tpt1* mutant, 2’-phosphorylated tRNAs accumulate, and the tRNAs show altered posttranscriptional modification (Spinelli *et al.* 1997).

##### CPD1:

Each tRNA splicing reaction produces an equimolar amount of ADP-ribose 1′′,2′′-cyclic phosphate (Appr > p). This molecule is converted into ADP-ribose-1′′-phosphate (Appr1p) by Cpd1 (Shull *et al.* 2005). It is estimated that ∼500 000 tRNA splicing events take place per *Saccharomyces cerevisiae* generation (Shull *et al.* 2005).

The significance of having found these two related enzymes in our screen is not clear. One possibility is that Appr1p or its derivatives may serve as signaling molecules in the cell, and an increase in its level may somehow be beneficial. We note that Tpt1 was identified as a histone H3-interacting protein (Gilmore *et al.* 2012).

#### Transcription factors:

The genes in this category are as follows:

##### CST6:

*CST6* was identified in a search for genes that are important for chromosome stability (CST genes) (Ouspenski *et al.* 1999). Interestingly, this screen also identified, among others, *PAB1*, as well as ubiquitin and histone genes (Ouspenski *et al.* 1999). Cst6 is a transcription factor activator, a member of the *S. cerevisiae* ATF/CREB family (Garcia-Gimeno and Struhl 2000). Its targets are still unknown.

##### RFX1:

RFX1 encodes the major transcriptional repressor of some DNA-damage-regulated genes. It helps recruit the repressors Tup1 and Cyc8 to the promoters of the relevant genes, such as the subunits of the ribonucleotide reductase enzyme. Upon DNA damage Rfx1 becomes phosphorylated and is released from the genes, allowing the recruitment of SAGA or TFIID subunits required for their expression (Ghosh and Pugh 2011).

##### TAF3:

Taf3 is a subunit of the TFIID complex, required for gene expression. It has lately been found that TFIID is preferentially recruited to promoters that lack TATA boxes, whereas SAGA binds preferentially TATA-containing regulatory sequences (Rhee and Pugh 2012).

The detection of these genes, involved in transcriptional regulation, as suppressors of the SL phenotype of *elg1Δ* and *srs2Δ*, implies that the suppression is possible by transcriptional activation or repression of target genes. However, the identity of the regulated genes, albeit, is still to be found.

#### Additional genes:

##### BAP2:

Bap2 encodes a high-affinity leucine permease that can also function as a branched-chain amino acid permease involved in the uptake of isoleucine and valine (Grauslund *et al.* 1995).

##### SRL3:

The *SRL3* gene was identified in a screen for high copy number suppressors of the essential function of the DNA damage response genes *MEC1* (ATR in humans) and *RAD53* (CHK2) (Desany *et al.* 1998). Its exact function remains enigmatic.

##### IRC9, IRC13:

These two genes were identified in a screen for mutants exhibiting increased levels of Rad52 foci. Both are considered “dubious ORFs” by the Saccharomyces Gene Database (http://www.yeastgenome.org).

##### YOL114c:

*YOL114c* is an uncharacterized ORF.

##### YKL044w:

This small ORF is present within the UTR of the *PHD1* gene. Evidence for its being translated comes from ribosomal profiling experiments (Brar *et al.* 2012).

In summary, we have carried out a screen for genes that, when overexpressed, can suppress the SL of an *elg1Δ srs2Δ* strain. We did not isolate plasmids carrying the *ELG1* or *SRS2* genes; however, this is an expected result: overexpression of these genes is slightly toxic to the cells, and these genes tend to be underrepresented when screening high copy number libraries (Leon Ortiz *et al.* 2011; Parnas *et al.* 2009). The coverage used (>x20) allowed us to identify a relatively large number of candidate genes, which were confirmed one by one by re-cloning and re-testing. Although most genes were identified only once, several of the genomic regions were independently isolated several times, and some (*e.g.*, *PAB1*) were identified six times. Thus, we are confident that our results represent a significant fraction of the genes that affect the genetic interactions between *SRS2* and *ELG1*. It appears that there is a large number of ways in which the SL phenotype can be bypassed. Our results suggest that chromatin modification and sister chromatid cohesion are processes related to the function defective in *elg1Δ srs2Δ* double mutants. In addition, increased transcription/translation and nuclear export seem to be able to bypass the synthetic phenotype, possibly by affecting an effector protein(s). We believe that the results obtained from this overexpression screen will provide valuable information for further research needed to better understand the functions carried out by these genes and their interactions.

## References

[bib1] Acharya N. Yoon J. H. Gali H. Unk I. Haracska L., 2008 Roles of PCNA-binding and ubiquitin-binding domains in human DNA polymerase eta in translesion DNA synthesis. Proc. Natl. Acad. Sci. USA 105: 17724–17729 19001268 10.1073/pnas.0809844105 PMC2584706

[bib2] Agmon N. Pur S. Liefshitz B. Kupiec M., 2009 Analysis of repair mechanism choice during homologous recombination. Nucleic Acids Res. 37: 5081–5092 19553188 10.1093/nar/gkp495 PMC2731894

[bib3] Aitchison J. D. Rout M. P., 2012 The yeast nuclear pore complex and transport through it. Genetics 190: 855–883 22419078 10.1534/genetics.111.127803 PMC3296253

[bib4] Almedawar S. Colomina N. Bermudez-Lopez M. Pocino-Merino I. Torres-Rosell J., 2012 A SUMO-dependent step during establishment of sister chromatid cohesion. Curr. Biol. 22: 1576–1581 22771040 10.1016/j.cub.2012.06.046

[bib5] Amerik A. Swaminathan S. Krantz B. A. Wilkinson K. D. Hochstrasser M., 1997 In vivo disassembly of free polyubiquitin chains by yeast Ubp14 modulates rates of protein degradation by the proteasome. EMBO J. 16: 4826–4838 9305625 10.1093/emboj/16.16.4826 PMC1170118

[bib6] Amrani N. Minet M. Le Gouar M. Lacroute F. Wyers F., 1997 Yeast Pab1 interacts with Rna15 and participates in the control of the poly(A) tail length in vitro. Mol. Cell. Biol. 17: 3694–3701 9199303 10.1128/mcb.17.7.3694 PMC232221

[bib7] Amrani N. Ghosh S. Mangus D. A. Jacobson A., 2008 Translation factors promote the formation of two states of the closed-loop mRNP. Nature 453: 1276–1280 18496529 10.1038/nature06974 PMC2587346

[bib8] Armstrong A. A. Mohideen F. Lima C. D., 2012 Recognition of SUMO-modified PCNA requires tandem receptor motifs in Srs2. Nature 483: 59–63 22382979 10.1038/nature10883 PMC3306252

[bib9] Aylon Y. Liefshitz B. Bitan-Banin G. Kupiec M., 2003 Molecular dissection of mitotic recombination in the yeast Saccharomyces cerevisiae. Mol. Cell. Biol. 23: 1403–1417 12556499 10.1128/MCB.23.4.1403-1417.2003 PMC141147

[bib10] Banerjee S. Myung K., 2004 Increased genome instability and telomere length in the elg1-deficient *Saccharomyces cerevisiae* mutant are regulated by S-phase checkpoints. Eukaryot. Cell 3: 1557–1566 15590829 10.1128/EC.3.6.1557-1566.2004 PMC539025

[bib11] Banerjee S. Sikdar N. Myung K., 2007 Suppression of gross chromosomal rearrangements by a new alternative replication factor C complex. Biochem. Biophys. Res. Commun. 362: 546–549 17689491 10.1016/j.bbrc.2007.07.126 PMC2034446

[bib12] Bellaoui M. Chang M. Ou J. Xu H. Boone C., 2003 Elg1 forms an alternative RFC complex important for DNA replication and genome integrity. EMBO J. 22: 4304–4313 12912927 10.1093/emboj/cdg406 PMC175796

[bib13] Ben-Aroya S. Koren A. Liefshitz B. Steinlauf R. Kupiec M., 2003 ELG1, a yeast gene required for genome stability, forms a complex related to replication factor C. Proc. Natl. Acad. Sci. USA 100: 9906–9911 12909721 10.1073/pnas.1633757100 PMC187881

[bib14] Brachat A. Kilmartin J. V. Wach A. Philippsen P., 1998 *Saccharomyces cerevisiae* cells with defective spindle pole body outer plaques accomplish nuclear migration via half-bridge-organized microtubules. Mol. Biol. Cell 9: 977–991 9571234 10.1091/mbc.9.5.977 PMC25323

[bib15] Branzei D. Vanoli F. Foiani M., 2008 SUMOylation regulates Rad18-mediated template switch. Nature 456: 915–920 19092928 10.1038/nature07587

[bib16] Brar G. A. Yassour M. Friedman N. Regev A. Ingolia N. T., 2012 High-resolution view of the yeast meiotic program revealed by ribosome profiling. Science 335: 552–557 22194413 10.1126/science.1215110 PMC3414261

[bib17] Bustard D. E. Menolfi D. Jeppsson K. Ball L. G. Dewey S. C., 2012 During replication stress, non-SMC element 5 (NSE5) is required for Smc5/6 protein complex functionality at stalled forks. J. Biol. Chem. 287: 11374–11383 22303010 10.1074/jbc.M111.336263 PMC3322872

[bib18] Chavez A. Agrawal V. Johnson F. B., 2011 Homologous recombination-dependent rescue of deficiency in the structural maintenance of chromosomes (Smc) 5/6 complex. J. Biol. Chem. 286: 5119–5125 21138837 10.1074/jbc.M110.201608 PMC3037623

[bib19] Chen Y. H. Choi K. Szakal B. Arenz J. Duan X., 2009 Interplay between the Smc5/6 complex and the Mph1 helicase in recombinational repair. Proc. Natl. Acad. Sci. USA 106: 21252–21257 19995966 10.1073/pnas.0908258106 PMC2795505

[bib20] Chilkova O. Stenlund P. Isoz I. Stith C. M. Grabowski P., 2007 The eukaryotic leading and lagging strand DNA polymerases are loaded onto primer-ends via separate mechanisms but have comparable processivity in the presence of PCNA. Nucleic Acids Res. 35: 6588–6597 17905813 10.1093/nar/gkm741 PMC2095795

[bib21] Chiolo I. Carotenuto W. Maffioletti G. Petrini J. H. Foiani M., 2005 Srs2 and Sgs1 DNA helicases associate with Mre11 in different subcomplexes following checkpoint activation and CDK1-mediated Srs2 phosphorylation. Mol. Cell. Biol. 25: 5738–5751 15964827 10.1128/MCB.25.13.5738-5751.2005 PMC1156977

[bib22] Clemente-Ruiz M. Gonzalez-Prieto R. Prado F., 2011 Histone H3K56 acetylation, CAF1, and Rtt106 coordinate nucleosome assembly and stability of advancing replication forks. PLoS Genet. 7: e1002376 22102830 10.1371/journal.pgen.1002376 PMC3213180

[bib23] Cocklin R. Heyen J. Larry T. Tyers M. Goebl M., 2011 New insight into the role of the Cdc34 ubiquitin-conjugating enzyme in cell cycle regulation via Ace2 and Sic1. Genetics 187: 701–715 21196523 10.1534/genetics.110.125302 PMC3063666

[bib24] Costanzo M. Baryshnikova A. Bellay J. Kim Y. Spear E. D., 2010 The genetic landscape of a cell. Science 327: 425–431 20093466 10.1126/science.1180823 PMC5600254

[bib25] Culver G. M. McCraith S. M. Consaul S. A. Stanford D. R. Phizicky E. M., 1997 A 2’-phosphotransferase implicated in tRNA splicing is essential in Saccharomyces cerevisiae. J. Biol. Chem. 272: 13203–13210 9148937 10.1074/jbc.272.20.13203

[bib26] Daee D. L. Ferrari E. Longerich S. Zheng X. F. Xue X., 2012 Rad5-dependent DNA repair functions of the Saccharomyces cerevisiae FANCM protein homolog Mph1. J. Biol. Chem. 287: 26563–26575 22696213 10.1074/jbc.M112.369918 PMC3410997

[bib27] Davidson M. B. Katou Y. Keszthelyi A. Sing T. L. Xia T., 2012 Endogenous DNA replication stress results in expansion of dNTP pools and a mutator phenotype. EMBO J. 31: 895–907 22234187 10.1038/emboj.2011.485 PMC3280564

[bib28] de Nadal E. Clotet J. Posas F. Serrano R. Gomez N., 1998 The yeast halotolerance determinant Hal3p is an inhibitory subunit of the Ppz1p Ser/Thr protein phosphatase. Proc. Natl. Acad. Sci. USA 95: 7357–7362 9636153 10.1073/pnas.95.13.7357 PMC22616

[bib29] Desany B. A. Alcasabas A. A. Bachant J. B. Elledge S. J., 1998 Recovery from DNA replicational stress is the essential function of the S-phase checkpoint pathway. Genes Dev. 12: 2956–2970 9744871 10.1101/gad.12.18.2956 PMC317167

[bib30] Dotiwala F. Harrison J. C. Jain S. Sugawara N. Haber J. E., 2010 Mad2 prolongs DNA damage checkpoint arrest caused by a double-strand break via a centromere-dependent mechanism. Curr. Biol. 20: 328–332 20096585 10.1016/j.cub.2009.12.033 PMC2811853

[bib31] Dover J. Schneider J. Tawiah-Boateng M. A. Wood A. Dean K., 2002 Methylation of histone H3 by COMPASS requires ubiquitination of histone H2B by Rad6. J. Biol. Chem. 277: 28368–28371 12070136 10.1074/jbc.C200348200

[bib32] Dunn E. F. Hammell C. M. Hodge C. A. Cole C. N., 2005 Yeast poly(A)-binding protein, Pab1, and PAN, a poly(A) nuclease complex recruited by Pab1, connect mRNA biogenesis to export. Genes Dev. 19: 90–103 15630021 10.1101/gad.1267005 PMC540228

[bib33] Ede C. Rudolph C. J. Lehmann S. Schurer K. A. Kramer W., 2011 Budding yeast Mph1 promotes sister chromatid interactions by a mechanism involving strand invasion. DNA Repair (Amst.) 10: 45–55 20951099 10.1016/j.dnarep.2010.09.009

[bib34] Ernsting B. R. Dixon J. E., 1997 The PPS1 gene of *Saccharomyces cerevisiae* codes for a dual specificity protein phosphatase with a role in the DNA synthesis phase of the cell cycle. J. Biol. Chem. 272: 9332–9343 9083070 10.1074/jbc.272.14.9332

[bib35] Garcia-Gimeno M. A. Struhl K., 2000 Aca1 and Aca2, ATF/CREB activators in *Saccharomyces cerevisiae*, are important for carbon source utilization but not the response to stress. Mol. Cell. Biol. 20: 4340–4349 10825197 10.1128/mcb.20.12.4340-4349.2000 PMC85801

[bib36] Ghosh S. Pugh B. F., 2011 Sequential recruitment of SAGA and TFIID in a genomic response to DNA damage in *Saccharomyces cerevisiae*. Mol. Cell. Biol. 31: 190–202 20956559 10.1128/MCB.00317-10 PMC3019861

[bib37] Gilmore J. M. Sardiu M. E. Venkatesh S. Stutzman B. Peak A., 2012 Characterization of a highly conserved histone related protein, Ydl156w, and its functional associations using quantitative proteomic analyses. Mol. Cell. Proteomics. 11: M111:011544 22199229 10.1074/mcp.M111.011544 PMC3322567

[bib38] Grauslund M. Didion T. Kielland-Brandt M. C. Andersen H. A., 1995 BAP2, a gene encoding a permease for branched-chain amino acids in *Saccharomyces cerevisiae*. Biochim. Biophys. Acta 1269: 275–280 7495881 10.1016/0167-4889(95)00138-8

[bib39] Harel-Sharvit L. Eldad N. Haimovich G. Barkai O. Duek L., 2010 RNA polymerase II subunits link transcription and mRNA decay to translation. Cell 143: 552–563 21074047 10.1016/j.cell.2010.10.033

[bib40] Hoege C. Pfander B. Moldovan G. L. Pyrowolakis G. Jentsch S., 2002 RAD6-dependent DNA repair is linked to modification of PCNA by ubiquitin and SUMO. Nature 419: 135–141 12226657 10.1038/nature00991

[bib41] Imbeault D. Gamar L. Rufiange A. Paquet E. Nourani A., 2008 The Rtt106 histone chaperone is functionally linked to transcription elongation and is involved in the regulation of spurious transcription from cryptic promoters in yeast. J. Biol. Chem. 283: 27350–27354 18708354 10.1074/jbc.C800147200

[bib42] Izaurralde E. Adam S., 1998 Transport of macromolecules between the nucleus and the cytoplasm. RNA 4: 351–364 9630243 PMC1369623

[bib43] Kanellis P. Agyei R. Durocher D., 2003 Elg1 forms an alternative PCNA-interacting RFC complex required to maintain genome stability. Curr. Biol. 13: 1583–1595 13678589 10.1016/s0960-9822(03)00578-5

[bib44] Karras G. I. Fumasoni M. Sienski G. Vanoli F. Branzei D., 2013 Noncanonical role of the 9-1-1 clamp in the error-free DNA damage tolerance pathway. Mol. Cell 49: 536–546 23260657 10.1016/j.molcel.2012.11.016

[bib45] Kim M. Krogan N. J. Vasiljeva L. Rando O. J. Nedea E., 2004 The yeast Rat1 exonuclease promotes transcription termination by RNA polymerase II. Nature 432: 517–522 15565157 10.1038/nature03041

[bib46] Klein H. L., 2001 Mutations in recombinational repair and in checkpoint control genes suppress the lethal combination of srs2Delta with other DNA repair genes in *Saccharomyces cerevisiae*. Genetics 157: 557–565 11156978 10.1093/genetics/157.2.557 PMC1461529

[bib47] Kolesar P. Sarangi P. Altmannova V. Zhao X. Krejci L., 2012 Dual roles of the SUMO-interacting motif in the regulation of Srs2 sumoylation. Nucleic Acids Res. 40: 7831–7843 22705796 10.1093/nar/gks484 PMC3439891

[bib48] Koren A. Ben-Aroya S. Steinlauf R. Kupiec M., 2003 Pitfalls of the synthetic lethality screen in *Saccharomyces cerevisiae*: an improved design. Curr. Genet. 43: 62–69 12684846 10.1007/s00294-003-0373-8

[bib49] Koshland D. Kent J. C. Hartwell L. H., 1985 Genetic analysis of the mitotic transmission of minichromosomes. Cell 40: 393–403 3881185 10.1016/0092-8674(85)90153-9

[bib50] Krejci L. Van Komen S. Li Y. Villemain J. Reddy M. S., 2003 DNA helicase Srs2 disrupts the Rad51 presynaptic filament. Nature 423: 305–309 12748644 10.1038/nature01577

[bib51] Krishna T. S. Kong X. P. Gary S. Burgers P. M. Kuriyan J., 1994 Crystal structure of the eukaryotic DNA polymerase processivity factor PCNA. Cell 79: 1233–1243 8001157 10.1016/0092-8674(94)90014-0

[bib52] Latham J. A. Chosed R. J. Wang S. Dent S. Y., 2011 Chromatin signaling to kinetochores: transregulation of Dam1 methylation by histone H2B ubiquitination. Cell 146: 709–719 21884933 10.1016/j.cell.2011.07.025 PMC3168986

[bib53] Lee J. S. Garrett A. S. Yen K. Takahashi Y. H. Hu D., 2012 Codependency of H2B monoubiquitination and nucleosome reassembly on Chd1. Genes Dev. 26: 914–919 22549955 10.1101/gad.186841.112 PMC3347789

[bib54] Lee K. Y. Myung K., 2008 PCNA modifications for regulation of post-replication repair pathways. Mol. Cells 26: 5–11 18525240 PMC3518309

[bib55] Leon Ortiz A. M. Reid R. J. Dittmar J. C. Rothstein R. Nicolas A., 2011 Srs2 overexpression reveals a helicase-independent role at replication forks that requires diverse cell functions. DNA Repair (Amst.) 10: 506–517 21459050 10.1016/j.dnarep.2011.02.004 PMC3084345

[bib56] Li Q. Burgess R. Zhang Z., 2012 All roads lead to chromatin: Multiple pathways for histone deposition. Biochim. Biophys. Acta 1819: 238–246 21763476 10.1016/j.bbagrm.2011.06.013 PMC3932183

[bib57] Liefshitz B. Parket A. Maya R. Kupiec M., 1995 The role of DNA repair genes in recombination between repeated sequences in yeast. Genetics 140: 1199–1211 7498763 10.1093/genetics/140.4.1199 PMC1206687

[bib58] Liefshitz B. Steinlauf R. Friedl A. Eckardt-Schupp F. Kupiec M., 1998 Genetic interactions between mutants of the ’error-prone’ repair group of Saccharomyces cerevisiae and their effect on recombination and mutagenesis. Mutat. Res. 407: 135–145 9637242 10.1016/s0921-8777(97)00070-0

[bib59] Mah A. S. Jang J. Deshaies R. J., 2001 Protein kinase Cdc15 activates the Dbf2-Mob1 kinase complex. Proc. Natl. Acad. Sci. USA 98: 7325–7330 11404483 10.1073/pnas.141098998 PMC34667

[bib60] Maradeo M. E. Skibbens R. V., 2009 The Elg1-RFC clamp-loading complex performs a role in sister chromatid cohesion. PLoS ONE 4: e4707 19262753 10.1371/journal.pone.0004707 PMC2650802

[bib61] Maradeo M. E. Skibbens R. V., 2010 Replication factor C complexes play unique pro- and anti-establishment roles in sister chromatid cohesion. PLoS ONE 5: e15381 21060875 10.1371/journal.pone.0015381 PMC2965161

[bib62] Mayer A. Heidemann M. Lidschreiber M. Schreieck A. Sun M., 2012 CTD tyrosine phosphorylation impairs termination factor recruitment to RNA polymerase II. Science 336: 1723–1725 22745433 10.1126/science.1219651

[bib63] Melo J. A. Cohen J. Toczyski D. P., 2001 Two checkpoint complexes are independently recruited to sites of DNA damage in vivo. Genes Dev. 15: 2809–2821 11691833 10.1101/gad.903501 PMC312815

[bib64] Menssen R. Schweiggert J. Schreiner J. Kusevic D. Reuther J., 2012 Exploring the topology of the Gid complex, the E3 ubiquitin ligase involved in catabolite-induced degradation of gluconeogenic enzymes. J. Biol. Chem. 287: 25602–25614 22645139 10.1074/jbc.M112.363762 PMC3408164

[bib65] Miranda J. J. De Wulf P. Sorger P. K. Harrison S. C., 2005 The yeast DASH complex forms closed rings on microtubules. Nat. Struct. Mol. Biol. 12: 138–143 15640796 10.1038/nsmb896

[bib66] Moldovan G. L. Pfander B. Jentsch S., 2006 PCNA controls establishment of sister chromatid cohesion during S phase. Mol. Cell 23: 723–732 16934511 10.1016/j.molcel.2006.07.007

[bib67] Munoz I. Simon E. Casals N. Clotet J. Arino J., 2003 Identification of multicopy suppressors of cell cycle arrest at the G1-S transition in Saccharomyces cerevisiae. Yeast 20: 157–169 12518319 10.1002/yea.938

[bib68] Ouspenski I. I. Elledge S. J. Brinkley B. R., 1999 New yeast genes important for chromosome integrity and segregation identified by dosage effects on genome stability. Nucleic Acids Res. 27: 3001–3008 10454593 10.1093/nar/27.15.3001 PMC148523

[bib69] Pan X. Ye P. Yuan D. S. Wang X. Bader J. S., 2006 A DNA integrity network in the yeast Saccharomyces cerevisiae. Cell 124: 1069–1081 16487579 10.1016/j.cell.2005.12.036

[bib70] Papouli E. Chen S. Davies A. A. Huttner D. Krejci L., 2005 Crosstalk between SUMO and ubiquitin on PCNA is mediated by recruitment of the helicase Srs2p. Mol. Cell 19: 123–133 15989970 10.1016/j.molcel.2005.06.001

[bib71] Parnas O. Zipin-Roitman A. Mazor Y. Liefshitz B. Ben-Aroya S., 2009 The ELG1 clamp loader plays a role in sister chromatid cohesion. PLoS ONE 4: e5497 19430531 10.1371/journal.pone.0005497 PMC2676507

[bib72] Parnas O. Zipin-Roitman A. Pfander B. Liefshitz B. Mazor Y., 2010 Elg1, an alternative subunit of the RFC clamp loader, preferentially interacts with SUMOylated PCNA. EMBO J. 29: 2611–2622 20571511 10.1038/emboj.2010.128 PMC2928695

[bib73] Pfander B. Moldovan G. L. Sacher M. Hoege C. Jentsch S., 2005 SUMO-modified PCNA recruits Srs2 to prevent recombination during S phase. Nature 436: 428–433 15931174 10.1038/nature03665

[bib74] Posas F. Camps M. Arino J., 1995 The PPZ protein phosphatases are important determinants of salt tolerance in yeast cells. J. Biol. Chem. 270: 13036–13041 7768897 10.1074/jbc.270.22.13036

[bib75] Regelmann J. Schule T. Josupeit F. S. Horak J. Rose M., 2003 Catabolite degradation of fructose-1,6-bisphosphatase in the yeast *Saccharomyces cerevisiae*: a genome-wide screen identifies eight novel GID genes and indicates the existence of two degradation pathways. Mol. Biol. Cell 14: 1652–1663 12686616 10.1091/mbc.E02-08-0456 PMC153129

[bib76] Rhee H. S. Pugh B. F., 2012 Genome-wide structure and organization of eukaryotic pre-initiation complexes. Nature 483: 295–301 22258509 10.1038/nature10799 PMC3306527

[bib77] Robzyk K. Recht J. Osley M. A., 2000 Rad6-dependent ubiquitination of histone H2B in yeast. Science 287: 501–504 10642555 10.1126/science.287.5452.501

[bib78] Rojas M. Farr G. W. Fernandez C. F. Lauden L. McCormack J. C., 2012 Yeast Gis2 and Its human ortholog CNBP are novel components of stress-induced RNP granules. PLoS ONE 7: e52824 23285195 10.1371/journal.pone.0052824 PMC3528734

[bib79] Rout M. P. Aitchison J. D. Suprapto A. Hjertaas K. Zhao Y., 2000 The yeast nuclear pore complex: composition, architecture, and transport mechanism. J. Cell Biol. 148: 635–651 10684247 10.1083/jcb.148.4.635 PMC2169373

[bib80] Ruiz A. Gonzalez A. Munoz I. Serrano R. Abrie J. A., 2009 Moonlighting proteins Hal3 and Vhs3 form a heteromeric PPCDC with Ykl088w in yeast CoA biosynthesis. Nat. Chem. Biol. 5: 920–928 19915539 10.1038/nchembio.243

[bib81] Ruone S. Rhoades A. R. Formosa T., 2003 Multiple Nhp6 molecules are required to recruit Spt16-Pob3 to form yFACT complexes and to reorganize nucleosomes. J. Biol. Chem. 278: 45288–45295 12952948 10.1074/jbc.M307291200

[bib82] Sammons M. A. Samir P. Link A. J., 2011 *Saccharomyces cerevisiae* Gis2 interacts with the translation machinery and is orthogonal to myotonic dystrophy type 2 protein ZNF9. Biochem. Biophys. Res. Commun. 406: 13–19 21277287 10.1016/j.bbrc.2011.01.086

[bib83] Scherrer T. Femmer C. Schiess R. Aebersold R. Gerber A. P., 2011 Defining potentially conserved RNA regulons of homologous zinc-finger RNA-binding proteins. Genome Biol. 12: R3 21232131 10.1186/gb-2011-12-1-r3 PMC3091301

[bib84] Schiestl R. H. Prakash S. Prakash L., 1990 The SRS2 suppressor of rad6 mutations of *Saccharomyces cerevisiae* acts by channeling DNA lesions into the RAD52 DNA repair pathway. Genetics 124: 817–831 2182387 10.1093/genetics/124.4.817 PMC1203974

[bib85] Shull N. P. Spinelli S. L. Phizicky E. M., 2005 A highly specific phosphatase that acts on ADP-ribose 1’’-phosphate, a metabolite of tRNA splicing in *Saccharomyces cerevisiae*. Nucleic Acids Res. 33: 650–660 15684411 10.1093/nar/gki211 PMC548356

[bib86] Silva A. C. Xu X. Kim H. S. Fillingham J. Kislinger T., 2012 The replication-independent histone H3–H4 chaperones HIR, ASF1, and RTT106 co-operate to maintain promoter fidelity. J. Biol. Chem. 287: 1709–1718 22128187 10.1074/jbc.M111.316489 PMC3265854

[bib87] Skibbens R. V. Corson L. B. Koshland D. Hieter P., 1999 Ctf7p is essential for sister chromatid cohesion and links mitotic chromosome structure to the DNA replication machinery. Genes Dev. 13: 307–319 9990855 10.1101/gad.13.3.307 PMC316428

[bib88] Spinelli S. L. Consaul S. A. Phizicky E. M., 1997 A conditional lethal yeast phosphotransferase (tpt1) mutant accumulates tRNAs with a 2’-phosphate and an undermodified base at the splice junction. RNA 3: 1388–1400 9404890 PMC1369580

[bib89] St Onge R. P. Mani R. Oh J. Proctor M. Fung E., 2007 Systematic pathway analysis using high-resolution fitness profiling of combinatorial gene deletions. Nat. Genet. 39: 199–206 17206143 10.1038/ng1948 PMC2716756

[bib90] Stillman D. J., 2010 Nhp6: a small but powerful effector of chromatin structure in Saccharomyces cerevisiae. Biochim. Biophys. Acta 1799: 175–180 20123079 10.1016/j.bbagrm.2009.11.010 PMC2818483

[bib91] Stoepel J. Ottey M. A. Kurischko C. Hieter P. Luca F. C., 2005 The mitotic exit network Mob1p-Dbf2p kinase complex localizes to the nucleus and regulates passenger protein localization. Mol. Biol. Cell 16: 5465–5479 16176976 10.1091/mbc.E05-04-0337 PMC1289394

[bib92] Su D. Hu Q. Li Q. Thompson J. R. Cui G., 2012 Structural basis for recognition of H3K56-acetylated histone H3–H4 by the chaperone Rtt106. Nature 483: 104–107 22307274 10.1038/nature10861 PMC3439842

[bib93] Tan A. L. Rida P. C. Surana U., 2005 Essential tension and constructive destruction: the spindle checkpoint and its regulatory links with mitotic exit. Biochem. J. 386: 1–13 15521820 10.1042/BJ20041415 PMC1134761

[bib94] Tkach J. M. Yimit A. Lee A. Y. Riffle M. Costanzo M., 2012 Dissecting DNA damage response pathways by analysing protein localization and abundance changes during DNA replication stress. Nat. Cell Biol. 14: 966–976 22842922 10.1038/ncb2549 PMC3434236

[bib95] Toth A. Ciosk R. Uhlmann F. Galova M. Schleiffer A., 1999 Yeast cohesin complex requires a conserved protein, Eco1p(Ctf7), to establish cohesion between sister chromatids during DNA replication. Genes Dev. 13: 320–333 9990856 10.1101/gad.13.3.320 PMC316435

[bib96] Tsvetanova N. G. Klass D. M. Salzman J. Brown P. O., 2010 Proteome-wide search reveals unexpected RNA-binding proteins in *Saccharomyces cerevisiae*. PLoS ONE 5: 9 10.1371/journal.pone.0012671 PMC2937035 20844764

[bib97] Ulrich H. D. Jentsch S., 2000 Two RING finger proteins mediate cooperation between ubiquitin-conjugating enzymes in DNA repair. EMBO J. 19: 3388–3397 10880451 10.1093/emboj/19.13.3388 PMC313941

[bib98] Unal E. Heidinger-Pauli J. M. Kim W. Guacci V. Onn I., 2008 A molecular determinant for the establishment of sister chromatid cohesion. Science 321: 566–569 18653894 10.1126/science.1157880

[bib99] Veaute X. Jeusset J. Soustelle C. Kowalczykowski S. C. Le Cam E., 2003 The Srs2 helicase prevents recombination by disrupting Rad51 nucleoprotein filaments. Nature 423: 309–312 12748645 10.1038/nature01585

[bib100] Warbrick E., 2000 The puzzle of PCNA’s many partners. Bioessays 22: 997–1006 11056476 10.1002/1521-1878(200011)22:11<997::AID-BIES6>3.0.CO;2-#

[bib101] Wilmes G. M. Bergkessel M. Bandyopadhyay S. Shales M. Braberg H., 2008 A genetic interaction map of RNA-processing factors reveals links between Sem1/Dss1-containing complexes and mRNA export and splicing. Mol. Cell 32: 735–746 19061648 10.1016/j.molcel.2008.11.012 PMC2644724

